# Characterization of a splice-site mutation in the tumor suppressor gene *FLCN* associated with renal cancer

**DOI:** 10.1186/s12881-017-0416-5

**Published:** 2017-05-12

**Authors:** Malte P. Bartram, Tripti Mishra, Nadine Reintjes, Francesca Fabretti, Hakam Gharbi, Alexander C. Adam, Heike Göbel, Mareike Franke, Bernhard Schermer, Stefan Haneder, Thomas Benzing, Bodo B. Beck, Roman-Ulrich Müller

**Affiliations:** 1 Department II of Internal Medicine and Center for Molecular Medicine Cologne, University of Cologne, Kerpener Str. 62, 50937 Cologne, Germany; 2 Institute of Human Genetics, University of Cologne, Kerpener Str. 62, 50937 Cologne, Germany; 3 Department of Pathology, University of Cologne, Kerpener Str. 62, 50937 Cologne, Germany; 4 Department of Radiology, University of Cologne, Kerpener Str. 62, 50937 Cologne, Germany; 5 Dr. Hancken Clinic, Harsefelder Str. 8, 21680 Stade, Germany; 6 Cologne Excellence Cluster on Cellular Stress Responses in Aging-Associated Diseases (CECAD), University of Cologne, Cologne, Germany; 7 Systems Biology of Ageing Cologne (Sybacol), University of Cologne, Cologne, Germany

**Keywords:** FLCN, Folliculin, BHD syndrome, Birt-Hogg-Dubé syndrome, Kidney cancer, Renal cell carcinoma, Proteasome, Lysosome

## Abstract

**Background:**

Renal cell carcinoma is among the most prevalent malignancies. It is generally sporadic. However, genetic studies of rare familial forms have led to the identification of mutations in causative genes such as *VHL* and *FLCN*. Mutations in the *FLCN* gene are the cause of Birt-Hogg-Dubé syndrome, a rare tumor syndrome which is characterized by the combination of renal cell carcinoma, pneumothorax and skin tumors.

**Methods:**

Using Sanger sequencing we identify a heterozygous splice-site mutation in *FLCN* in lymphocyte DNA of a patient suffering from renal cell carcinoma. Furthermore, both tumor DNA and DNA from a metastasis are analyzed regarding this mutation. The pathogenic effect of the sequence alteration is confirmed by minigene assays and the biochemical consequences on the protein are examined using TALEN-mediated transgenesis in cultured cells.

**Results:**

Here we describe an *FLCN* mutation in a 55-year-old patient who presented himself with progressive weight loss, bilateral kidney cysts and renal tumors. He and members of his family had a history of recurrent pneumothorax during the last few decades. Histology after tumor nephrectomy showed a mixed kidney cancer consisting of elements of a chromophobe renal cell carcinoma and dedifferentiated small cell carcinoma component. Subsequent *FLCN* sequencing identified an intronic c.1177-5_-3delCTC alteration that most likely affected the correct splicing of exon 11 of the *FLCN* gene. We demonstrate skipping of exon 11 to be the consequence of this mutation leading to a shift in the reading frame and the insertion of a premature stop codon. Interestingly, the truncated protein was still expressed both in cell culture and in tumor tissue, though it was strongly destabilized and its subcellular localization differed from wild-type FLCN. Both, altered protein stability and subcellular localization could be partly reversed by blocking proteasomal and lysosomal degradation.

**Conclusions:**

Identification of disease-causing mutations in BHD syndrome requires the analysis of intronic sequences. However, biochemical validation of the consecutive alterations of the resulting protein is especially important in these cases. Functional characterization of the disease-causing mutations in BHD syndrome may guide further research for the development of novel diagnostic and therapeutic strategies.

**Electronic supplementary material:**

The online version of this article (doi:10.1186/s12881-017-0416-5) contains supplementary material, which is available to authorized users.

## Background

Birt-Hogg-Dubé syndrome (BHD) is a rare autosomal dominant inherited tumor syndrome caused by mutations in the *FLCN* gene [1, 2]. The disease is characterized by a triad of renal cell carcinoma, benign skin tumors (fibrofolliculomas) and pulmonary cysts that predispose to recurrent pneumothorax. Since the clinical presentation is highly variable even within families, it is likely that BHD syndrome is an underdiagnosed disorder. About 600 families with *FLCN* mutations have been reported. Increasing awareness of the disease results in a steadily growing number of diagnoses [3–5]. The prevalence of the clinically most important consequence of BHD syndrome, renal cell carcinoma, varies between studies from 12 to 34% [6, 7]. In line with Knudson’s hypothesis loss of heterozygosity or somatic mutations have been observed in renal tumor tissue [8, 9]. Due to the strongly increased risk of renal cell carcinoma in patients with BHD syndrome and the occurrence of renal tumors in rodent models of the disease FLCN can be considered a tumor suppressor protein. On the molecular level, FLCN has been linked to a multitude of pathways including mTOR, AMPK signaling, the VHL/HIF/VEGF axis, TGFb signaling, autophagy and cilia (reviewed in [10]; [11–20]) although the exact function is incompletely understood and is subject to intense research. Currently 159 disease causing FLCN mutations have been reported in the Human Gene Mutation Database (HGMD professional; assessed 2016.4; https://portal.biobase-international.com/hgmd/pro/). Matchable numbers can be obtained from a publicly available database - the Folliculin Sequence Variation Database [4] - hosted online by the Leiden Open Variation Database (LOVD), which currently contains 152 FLCN mutations; data freeze January 2017; https://www.bhdsyndrome.org/). Small deletions or duplications, small insertions and small insertions/deletions (indels) that predominantly result in a frameshift (*n* = 80; ca. 50%, all numbers here refer to HGMD), nonsense mutations (*n* = 23; ca. 15%), gross (genomic) deletions or amplifications (*n* = 22; ca. 14%) and (intronic) splice mutations (*n* = 21; ca. 13%) account for the vast majority of cases (ca. 92%), while missense mutations are comparatively rare (*n* = 13; ca. 8%). The reported frequency of gross (genomic) deletions/amplifications in BHD makes MLPA analyses (multiplex ligation-dependent probe amplification) a useful tool in the diagnostic work of suspected BHD cases. Whilst the consequences of sequence alterations in the coding regions are often obvious, the pathogenic potential of many splice mutations and (intronic) variants likely affecting splicing found in BHD syndrome - including c.1177-5_-3delCTC - has not been studied well beyond in silico analyses [4, 5, 21].

## Methods

### Sanger sequencing

After obtaining written informed consent from the index patient, mutational analysis of the *FLCN* gene was performed by exon PCR of all 11 coding exons. Primer sequences for the exons and adjacent intron-exon borders were designed with the Exon Primer software (https://ihg.helmholtz-muenchen.de/ihg/ExonPrimer.html). For analyses of formalin-fixed paraffin-embedded tissue sections primers generating appropriate short amplicons (150 bp) were selected. Primer sequences and PCR conditions are available on request. Purified PCR products were sequenced by BigDye terminator ready reaction kit v.1.1 (Life Technologies), using a 3500 Genetic Analyzer (Applied Biosystems). Resulting sequences were evaluated with the Sequence Pilot ™ software (JSI Medical Systems GmbH, Germany).

### Cell lines

HEK293T (gift from Brian Seed (Boston)), UOK257 (gift from Laura Schmidt and Michael Pollak (NIH)) and IMCD (from ATCC) cells were maintained in DMEM + 10% fetal bovine serum (FBS) at 37 °C. RPE-1 cells (from ATCC) were maintained in DMEM/F12 + 10% fetal bovine serum (FBS) at 37 °C.

### Construction of FLCN expression plasmids, minigenes and minigene assay

To generate expression plasmids of FLCN WT PCRs using sequence specific primers that generate a 5′ Mlu1 site and 3′Not1 site to clone the PCR products into a modified pcDNA6 vector (Invitrogen) were used using standard techniques (fp: 5′-cgcgggacgcgtATGAATGCCATCGTGGCTCTC-3′; rp 5′-gcggggcggccgccTCAGTTCCGAGACTCCGAGGC-3′). The plasmid expressing the FLCN patient mutation (missing exon 11) was generated using overlap extension PCRs. All constructs were verified by sequencing.

The minigene-11 construct containing parts of human FLCN exon 10, intron 10 and parts of exon 11 was cloned via PCR using a forward primer that is located in exon 10 and a reverse primer located in exon 11 into a modified pcDNA6 vector (Invitrogen). The minigene-2 construct, containing parts of exon 10, intron 10, exon11, intron 11 and parts of exon 12 a forward primer located in exon 10 and a reverse primer located in exon 12 was used. Both, the WT and the patient mutation constructs were cloned by PCR using patient genomic DNA derived from lymphocytes as a template. WT and mutant clones were identified by Sanger Sequencing. All plasmids were verified by sequencing. Afterwards the plasmids were transfected into mouse cells (IMCD) to prevent that endogenous human FLCN interferes with the analysis of the minigenes. 24 h after transfection RNA was extracted from the cells using standard methods and cDNA was generated (ABI HighCapacity cDNA Kit). Afterwards PCRs were performed and the PCR products resolved on an agarose gel (primers for minigene-1: fp 5′- tgggtgccccttctttc rp 5′- gctgagccccaggaagtt; primers for minigene-2: fp 5′- aaccaggtgatctggaaa rp 5′- cactggtcaccacaaactcg. The indicated bands/PCR products were further analysed by Sanger sequencing.

### AAV-CAGGS-EGFP-hFLCN transfection in HEK293T cells to generate TALEN cells

The sequences of FLCN WT and the patient mutation missing exon11 were subcloned into a modified AAV-CAGGS-EGFP vector (a TALEN-based vector, Addgene 22212) in a way that a GFP fusion protein is created. The constructs were confirmed by sequencing. HEK293T cells were transfected with 1 μg AAV-CAGGS-EGFP-hFLCN WT or 1 μg AAV-CAGGS-EGFP-hFLCN Mut) along with 0.5 μg hAAVS1 1 L TALEN and 0.5 μg hAAVS1 1 TALEN nuclease plasmids using the calcium phosphate transfection method. 48 h after transfection, cells were kept in 2 μg puromycin selection. Few colonies were found after selection for 2 weeks. The colonies were then expanded and further analysed by Western Blot and imaging experiments.

### MG-132 treatment and chloroquine treatment

Cells were treated with 10 μM MG-132 (Calbiochem, cat. # 474790) for 2 h and 50 μM Chloroquine (Sigma, cat. # C6628) for 24 h respectively. After treatment, cells were harvested in 10 ml ice-cold PBS and then centrifuged at 200 *g* for 5 min at 4 °C. The supernatant was discarded and the pellet was resuspended in 300 μl IP buffer (1% Triton-X-100, 20 mM Tris HCl pH 7.5, 50 mM NaCl, 25 mM NaF, 12.5 mM Na_4_P_2_O_7,_ 2 mM Na_3_VO_4,_ PMSF (44 μl/10 ml)) and incubated for 15 min on ice. Lysates were then centrifuged at 20800 *g* for 30 min at 4 °C. 200 μl supernatant was transferred to a new 1.5 ml tube for further use. 40 μl of this supernatant was taken and boiled with 40 μl 2× Laemmli buffer at 95 °C for 5 min followed by Western blot analyses (mouse anti-GFP (St. Cruz, sc-9996) and mouse anti-Tubulin (clone E7, DSHB).

### Fluorescence imaging

Stably integrated AAV-CAGGS-EGFP-hFLCN WT and AAV-CAGGS-EGFP-hFLCN Mut cells were fixed in 4% PFA for 15 min at room temperature. PFA was discarded and cells were washed 3 times with PBS (supplemented with Ca^2+^ and Mg^2+^). Mounting was performed using Prolong Gold with DAPI (Invitrogen). Images were visualized using Axiovert 200 microscope (objective: C-Apochromat × 63/1.22 W).

### siRNA experiments

RPE-1 cells were transfected using Lipofectamine 2000 (Invitrogen) according to the manufacturer’s protocol with a final siRNA concentration of 25 nmol/μl. 21mer siRNA oligos were purchased from Biomers (Ulm, Germany). The following sequences were used: Control siRNA: AAATGTACtgcgcgtggagac; FLCN siRNA#1 auggugaugaugcuguacctt. The second FLCN siRNA was purchased from ThermoFisher Scientific (USA) as ready annealed siRNA duplex (J-009998-05-0005).

### Generation of cell lysates and Western Blot

Whole cell lysates of the RPE-1 cells transfected with the control or FLCN targeting siRNAs were lysed directly in SDS-PAGE sample buffer (1× Laemmli). Equal amounts of protein were resolved using were resolved by SDS-PAGE, blotted on to PVDF-membranes and visualized with enhanced chemiluminescence after incubation of the blots with the respective antibodies (rabbit anti-FLCN antibody (CellSignaling, #3697) and mouse anti-actin (Millipore, MAB1501R).

### Generation of UOK257 cells reexpressing FLCN WT

To reexpress FLCN in the FLCN deficient UOK257 cell line F9. FLCN WT was cloned using standard techniques into a modified pENTR1A vector (Invitrogen) and afterwards recombined into pLenti6.3 using the Gateway technology (Invitrogen). pLenti6.3-emGFP served as control. After virus production in HEK293T cells the UOK257 cells were transduced for 24 h and afterwards selected using blasticidin. To validate the FLCN antibody the FLCN and GFP expressing cell lines were seeded onto coverslips and IHC stained using the rabbit anti-FLCN antibody (CellSignaling, #3697).

### Immunohistochemical staining

Four μm sections were cut from paraffin embedded, formalin-fixed tissue blocks, mounted on silane coated slides, dried and deparaffinised by routine techniques in xylene and rehydrated in ethanol (100, 100, 90, 70, 50%). Sections were then washed in Tris–HCl buffer (pH 8.0) before incubation with primary antibody (anti-Folliculin, CellSignaling #3697, 1:20, pretreatment: citrate puffer (pH 6.0),). The incubation was performed over night at 4 °C. Labeling was detected semiautomatically by DAKO TechMate™ 500 Plus with the DAKO REAL™ Detection System using the streptavidine-biotin system according to manufacturer’s protocol (Dako REAL™ Biotinylated Secondary Antibodies: goat anti-mouse and anti-rabbit immunoglobulins, Dako REAL™ streptavidine peroxidase, Dako REAL™ AEC/H2O2 Substrate Solution, Dako REAL™ Blocking Solution, Dako REAL™ Buffer Kit). Sections were washed (3 times 5 min in PBS), counterstained with haematoxylin and finally mounted in Aquatex (Merck, Darmstadt, Germany).

## Results

The 55-year-old index patient presented to our emergency department in a reduced condition with night sweats, anemia, weight loss and progressive edema of both legs. In the medical history bilateral lesions of both kidneys had been known for about 20 years. These had been classified as angiomyolipomas due to their radiological aspects and led to the clinical diagnosis of oligosymptomatic tuberous sclerosis. Due to several episodes of spontaneous macroscopic hematuria suspicion of an increased risk of bleeding had prevented biopsies of the lesions so far. In addition, the patient reported several episodes of pneumothorax in the past as well as the occurrence of pneumothorax in other family members.

**Fig. 1:**
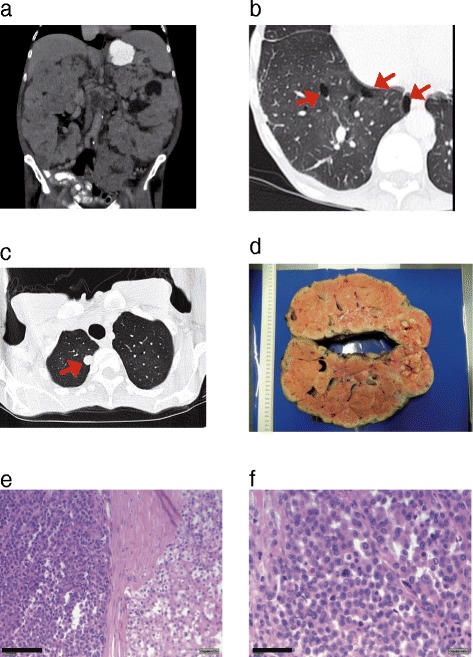
CT imaging of the patient and macroscopic and histopathological analysis of the kidney tumor tissue **a** CT scan of the abdomen shows massive enlarged kidneys with bilateral tumorous lesions. **b** and **c** CT scan of the thorax reveals numerous pulmonary cysts (**b**, *red arrows*) and several pulmonary lesions suspicious for metastastic disease (**c**, *red arrow*). **d** Macroscopic picture of the left kidney shows that the whole organ is interspersed with nodular tumors. **e** Beside the nodular growing chromophobe carcinoma (right side) a second tumor component consisting of very small tumor cells can be detected (left side of the panel, bar = 100 μm). **f** these small tumors cells show nuclear pleomorphism with increased and atypical mitosis (bar = 50 μm)

Cross-sectional imaging revealed dramatically enlarged kidneys (22 cm in length) with multiple tumorous lesions consistent with angiomyolipomas as well as an unclear 1.2x1.0 cm lesion on the lower pole of the left kidney (Fig. 1a). In addition several suspicious retroperitoneal lymph nodes were seen. Owing to their enormous size the kidneys compressed the pelvic vessels explaining the edema of the legs, echocardiography revealed a normal ejection fraction and no valvular disease. Furthermore, hepatosplenomegaly – most likely due to concurrent osteomyelofibrosis - and several lung cysts and pulmonary nodules were seen in a CT of the chest (Fig. 1b and c).

Nephrectomy of the left kidney was performed (Fig. 1d) to allow for histological examination of the lesions and relieve the compression of the inguinal vessels. Open surgery revealed peritoneal metastases. Pneumothorax of the left lung occurred as a perioperative complication and was treated with a chest tube. Histopathological work up of the renal tissue showed that the whole kidney was tumorous (Fig. 1d) with features of a chromophobe renal cell carcinoma (Fig. 1e (right side of the panel) and Additional file 1: Figure S1a-c). Beside the chromophobe carcinoma there was a second tumor entity consisting of small carcinoma cells with nuclear pleomorphism and atypical mitoses already invading the blood vessels (Fig. 1e (left side of the panel), Fig. 1f and Additional file 1: Figure S1c). Since the chromophobe carcinoma showed signs of dedifferentiation towards the small cell carcinoma component, we speculate that these cells might originate from the chRCC by acquiring further genetic alterations. Staining of several markers like CKAE1/3, CK7, TTF-1 and CD-*X*2 of the small cell carcinoma lesions were negative, indicating that these cells have no extrarenal origin (data not shown). There were no signs for lesions consistent with oncocytoma.

Histopathological work up of one peritoneal metastasis showed features of the small cellular tumor component (Additional file 1: Figure S1c and d), suggesting that these cells might contribute to the severe and aggressive tumor phenotype in our patient. Consistent with this observation, these cells showed a very high proliferation index analyzed by Ki-67 staining (Additional file 1: Figure S1f and g). Further immunohistological analysis of the metastastic tissue showed no staining for TTF-1, CD10 or ChrA (data not shown) as expected since the scattered small cell component that is seen throughout the tumor appears to be a less differentiated part of the chRCC. Soon after surgery hemodialysis was initiated due to end-stage renal failure. However, the patient died shortly afterwards as a consequence of the advanced stage of the metastatic tumor disease.

**Fig. 2:**
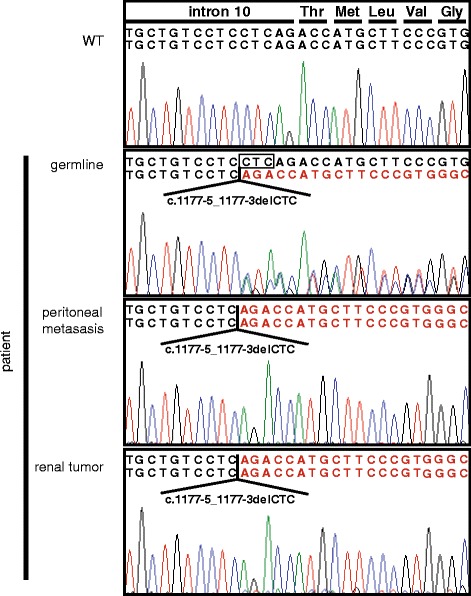
Sanger sequencing of lymphocyte DNA of the patient reveal a heterozygous three nucleotide deletion in an intron *right upstream* of exon 11 (c.1177-5_1177-3). The same mutation was found in homozygous state when the renal tumor or the peritoneal metastasis was sequenced

The co-occurrence of chromophobe renal cell carcinoma with familial recurrent pneumothorax made us suspect Birt-Hogg-Dubé syndrome in this family. Sanger sequencing of lymphocyte DNA was performed and revealed a previously reported heterozygous intronic three nucleotide deletion c.1177-5_-3delCTC just upstream of exon 11 (Fig. 2).

In silico analyses by several bioinformatic tools predicted this variant to be likely pathogenic. Splicing assessment using the alamut visual 2.7 indicated a significant weakening of the native acceptor site of exon 11, however neither generation of a novel acceptor site nor exon skipping were predicted by the EX SKIP tool contained in alamut visual [22]. Sanger sequencing of DNA extracted from PFA fixed renal tumor tissue and a peritoneal metastasis using primers adapted for short amplicons targeting the intron-exon border of exon 11, yielded c.1177-5_-3delCTC in an allegedly homozygous state. Since carcinogenesis in BHD syndrome is assumed to occur according to the Knudson’s hypothesis sequencing findings most likely represented loss-of-heterozygosity with deletion of the second *FLCN* allele in tumor tissue (Fig. 2). To further investigate this finding we performed array CGH and MLPA analyses. While array CGH on tumor material proved inconclusive on a 180 k standard array, *FLCN* MLPA analyses were consistent with deletion of the second *FLCN* allele in both tumor tissues (data not shown).

**Fig. 3:**
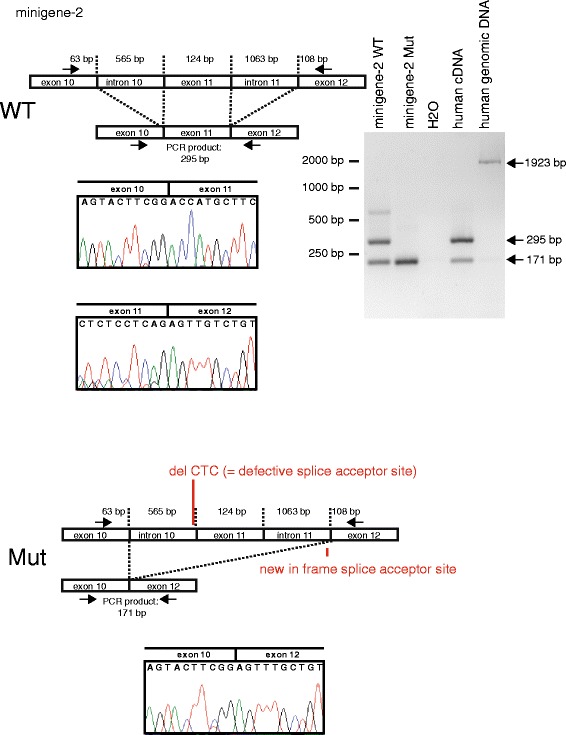
To investigate the consequence of the patient mutation, minigene-2 was engineered (containing exon 10, intron 10, exon 11, intron 11 and exon 12 of human *FLCN*). After transfection into mIMCD cells (of mouse origin) RNA and afterwards cDNA was prepared and subjected to sequencing and PCR. As expected, the PCR using the WT minigene-2 results (the fp and rp are located in exon 10 and exon 12, respectively) in a band of 295 bp (expected size after splicing of intron 10 (=565 bp) and intron 11 (=1063 bp)). Analysis of the mutant minigene-2 reveals that - as expected - in addition exon 11 (=124 bp) is spliced out resulting in a smaller PCR product of 171 bp. Sequencing of the PCR products reveals, that the defective splice acceptor site in front of exon11 leads to skipping of exon11. Since the splice acceptor site of exon 12 is in frame, fusion of exon 10 and exon 12 can be detected. Interestingly, also with the WT minigene-2 as well as with human cDNA skipping of exon 11 can be observed. Human cDNA and human genomic DNA served as controls for the PCR products. Sanger sequencings confirmed the results, electropherograms of the crucial junctions are shown

Since the mutation had been reported before in association with the clinical diagnosis of BHD syndrome [4, 23, 24] and the family in the latter report only presented with asymptomatic pneumothorax respectively lung cysts and skin manifestation we decided to further evaluate the effect of this sequence alteration to confirm its pathogenic potential on a molecular level. To validate the mutation’s impact on splicing of the FLCN transcript we generated minigene constructs containing either the *FLCN* wild-type (WT) or the c.1177-5—3 del CTC (Mut) sequence. These minigene constructs encoding parts of human *FLCN* were transfected into mouse IMCD cells to prevent that the amplification of endogenous human *FLCN* interferes with our analyses. Minigene construct 1 (minigene-1) consisted of parts of exon 10, intron 10-11 with (Mut) or without (WT) the mutation and parts of exon 11. The analysis revealed that the 3 bp deletion indeed abrogated the acceptor splice site of exon 11 (Additional file 2: Figure S2). To further explore how the mutation affects the splicing of the *FLCN* mRNA we used a second minigene (minigene-2) that consisted of exon 10, intron 10 (with and without the 3 bp deletion), exon 11, intron 11, and exon12. Sequencing of the respective bands showed that the intronic 3 bp deletion CTC at position -7 to -5 leads to skipping of exon 11 and fusion of exon 10 (in frame) to exon 12 which generates a frameshift and premature stop codon (p.T393Sfs33*) (Fig. 3). Interestingly, in the WT minigene-2 construct, exon 11 was differentially spliced and partially skipped to a certain degree as well. This could also be observed with human cDNA of endogenous FLCN. Yet, the full-length wildtype splice variant was never observed when expressing the mutated version of minigene- 2 (Fig. 3).

**Fig. 4:**
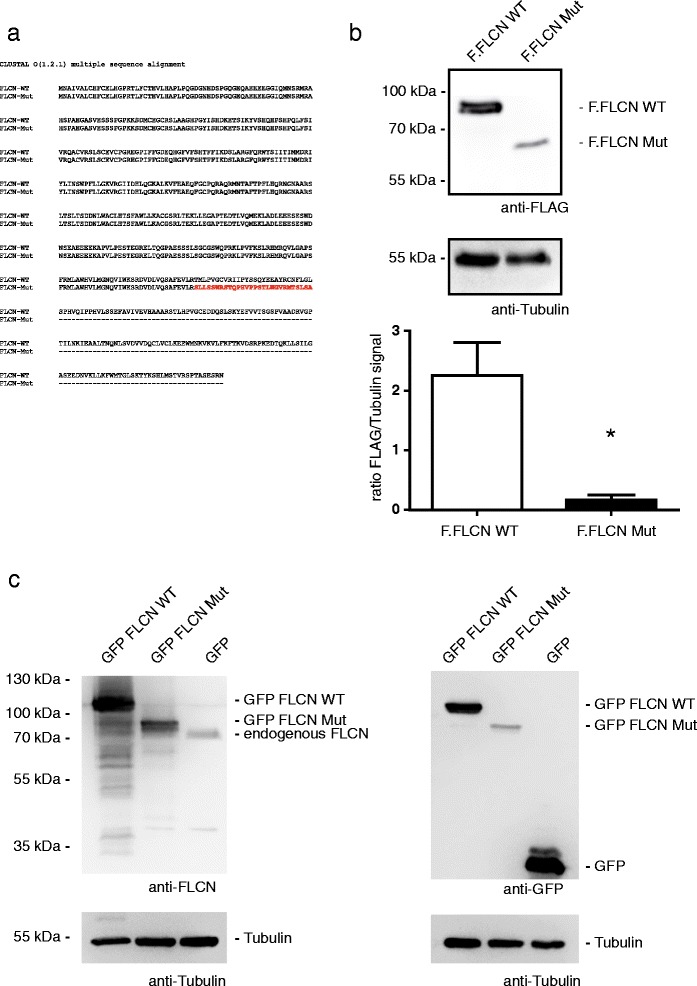
**a** Alignment of the FLCN WT and FLCN mutant sequence on the protein level. The altered amino acid sequence caused by the mutation is shown in *red*. The alignment was peformed using ClustalOmega [36]. **b** Equal amounts of FLAG tagged FLCN WT or Mut plasmids were transfected into HEK293T cells and the expression level of both constructs was analysed using Western Blot. The mutant protein (Mut) is less expressed in comparison to the WT protein. Tubulin served as loading control. The results of three independent experiments were analysed by densitometry (*n* =3, error bars indicate SEM, * = *p* < 0.05, two tailed *t*-test).**c** The FLCN antibody (CellSignaling, #3697) detects the FLCN mutant (delta exon 11). HEK293T cells were transfected with GFP tagged FLCN wildtype (WT), the patient mutation (Mut) or GFP (control). Western blot analyses show that the FLCN antibody detects both the FLCN WT and Mut protein as well as endogenous FLCN (panels on the left side). Expression of all plasmids was verified using an anti-GFP antibody. Tubulin served as loading control

To investigate whether the predicted FLCN neoprotein (p.T393Sfs33*, see Fig. 4a for an alignment of the protein sequences) is expressed at all we analyzed protein expression in HEK293T cells. Overexpression of a FLAG-tagged cDNA revealed that the mutant protein is expressed, however, at much lower levels than the WT protein (Fig. 4b and c). Since a commercially available anti-FLCN antibody (CellSignaling, #3697) detected the overexpressed mutant protein in Western Blots (Fig. 4c), we decided to use this antibody to analyze the endogenous expression in patient tumor tissue. SiRNA experiments and staining of the FLCN knockout cell line UOK257 as well as lentivirally transduced cells reexpressing FLCN revealed that the antibody specifically detected FLCN and could also be used for immune histochemistry (Additional file 3: Figure S3a and b). A distinct signal was obtained in the IHC analysis of the patient tumor indicating that expression of the mutant protein also occurs in vivo (Additional file 3: Figure S3c).

**Fig. 5:**
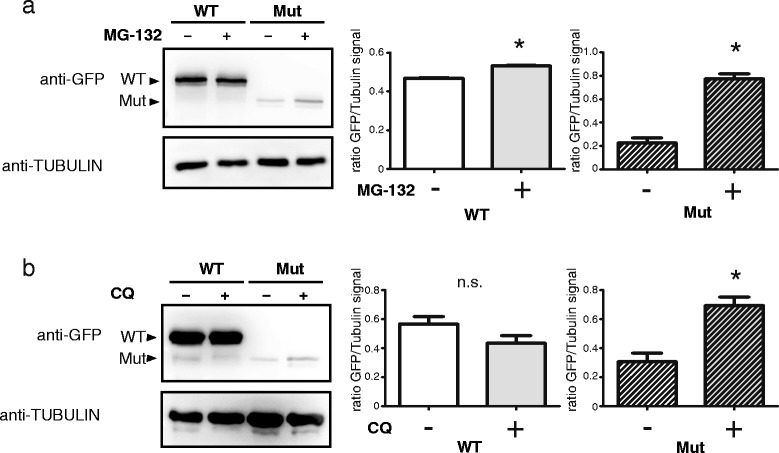
To analyse the impact of the patient mutation on the protein level, HEK293T cells expressing FLCN WT or Mut fused to GFP using the TALEN technology were generated. **a** and **b** Western blot analyses reveals that the mutant protein is hardly expressed. This could in part be alleviated with MG-132 (**a**, treatment with 10 μM for 2 h) or chloroquine (**b**, treatment with 50 μM for 24 h), indicating an involvement of the proteasome and lysosome in the degradation process. Tubulin served as loading control (*p* =3, error bars indicate SEM, * = *p* < 0.05, n.s. = not significant, two tailed *t*-test)

To confirm this finding we generated transgenic cell lines using the TALEN technology that express GFP-fused versions of either the WT or the mutant protein from the AAVS1-locus. This technology has the advantage of a single integration of the desired transgene into the genome, thereby resulting in expression levels that mimick the physiological situation more closely. Furthermore, since the transgenes were fused to GFP, imaging approaches are simplified. Whilst WT FLCN was easily detected by western blot the mutant protein again showed markedly lower protein levels (Fig. 5a and b). This effect could in part be reversed by treatment with MG-132 (Fig. 5a). Besides, treatment with chloroquine also increased the abundance of the mutant protein suggesting a combination of lysosomal and proteasomal degradation (Fig. 5b).

**Fig. 6:**
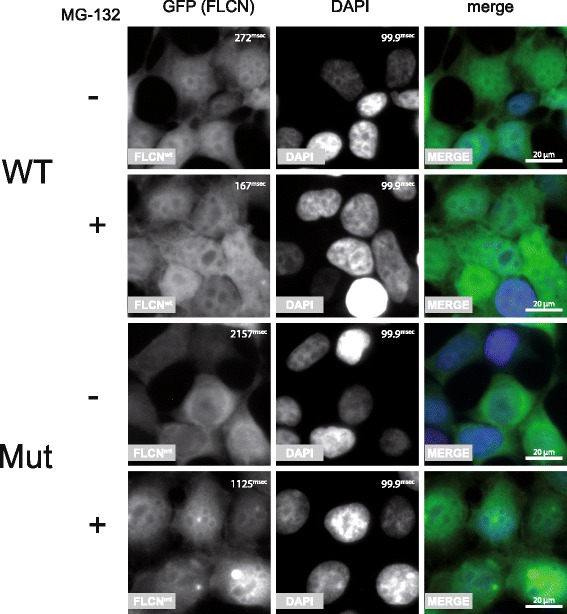
HEK293T cells expressing FLCN WT or Mut fused to GFP using the TALEN technology were subjected to immunofluorescent imaging. WT FLCN localizes to both cytoplasm and nucleus, the mutant protein is primarily found in the cytoplasm. Interestingly treatment with MG-132 did not only stabilize the mutant protein which is reflected by a shorter exposure time during image acquisition but also led to a nuclear shift making its localization more similar to the WT protein (bar = 20 μm, exposure time of the GFP and DAPI channel are depicted in millisecond in the upper right corner of each panel)

Additionally, fluorescence imaging revealed an altered subcellular localization profile of mutant FLCN in comparison to the WT protein. Whilst WT FLCN localizes to both cytoplasm and nucleus with a very distinct nuclear signal the mutant protein is primarily restricted to the cytoplasm. Interestingly, treatment with MG-132 did not only stabilize the mutant protein but also led to a nuclear shift (Fig. 6) making its localization more similar to the WT situation.

Taking into account the genetic exam as well as the biochemical confirmation of pathogenicity and the autosomal dominant nature of BHD syndrome we invited the kindred of our patient to genetic counseling and testing which revealed other members of his family showing features of BHD syndrome to carry the mutation.

## Discussion

Here we report a (familial) case of BHD with disseminated renal cell carcinoma and recurrent pneumothoraces in the index patient. Even though this can be regarded a classical picture of BHD syndrome there were a couple of unusual clinical as well as molecular aspects. First – even though present in about 90% of all BHD patients – physical examination results of the index patient did not describe a classical skin phenotype. Furthermore, in addition to the chromophobe RCC - the most common histology in BHD patients [25, 26] a poorly characterized small cell carcinoma was found upon histopathological analysis of the explanted kidney. BHD-syndrome associated renal cell carcinomas normally show a rather benign nature and rarely metastasize. Our patient suffered from both histologically confirmed peritoneal metastases and CT-morphological pulmonary lesions. The peritoneal metastases did not show the classical characteristics of the of the chromophobe RCC but rather resembled the less differentiated parts of this tumor showing a small-cell morphology. Since the chromophobe renal cell carcinoma showed different levels of dedifferentiation towards the areas of the small cell tumor component it is tempting to speculate that the small cell carcinoma arose from the chRCC by acquiring further genetic alterations. Furthermore, the metastasis appears to be linked to BHD syndrome since it showed loss-of-heterozygosity in the FLCN gene. It will be interesting to see in future cases whether this entity is associated with BHD syndrome. Hybrid tumors are a common feature in BHD patients, although most of these contain chromophobe and oncocytomic cancer tissue. Clear cell carcinomas (ccRCC) have been described as well [25]. In our case no oncocytomas or ccRCCs could be detected in the explanted kidney. Interestingly, to our knowledge a small cell carcinoma component had not previously described in tumors of BHD patients.

The mutation detected in our patient had been found in patients fulfilling at least a subset of clinical features of BHD syndrome before [23, 24]. However, whether the intronic variant observed really leads to a change in the resulting protein had not been proven on a biochemical level. Our findings emphasize the fact that mutations in non-coding regions have to be taken into account when analyzing genetic diseases and have probably been underestimated so far. While this manuscript was under revision a Danish group characterized the same mutation that they found in two﻿ sisters with RCC using a minigene assay and essentially found the same results in regard to splicing of WT and mutant FLCN [27].

As described above the mutant protein was expressed at much lower levels than the WT version. Previously it had been shown that the FLCN complex localizes to lysosomes and granules containing ubiquitin. Furthermore, the FLCN interaction partner FNIP is regulated by SCFβ-TRCP [20, 28, 29]. Inhibition of both lysosomal and proteasomal degradation stabilized the mutant protein. Additionally, MG-132 also partly rescued its localization defect. This may be the consequence of accumulation of ubiquitinated mutant FLCN, since ubiquitination has been shown to be a key regulator of subcellular localization of a number of different proteins [30–34]. Similar approaches have been hypothesized before as potential therapeutic strategies in genetic diseases [35]. Whether these approaches may have any therapeutic implication in the treatment of BHD – once stabilized and correctly localized - to fulfil the molecular function required to prevent tumorigenesis remains to be elucidated. Answering this question will require future studies in both cell culture and mouse models.

## Conclusions

Whilst most mutations identified in BHD syndrome so far affect the coding region it is to be expected that a significant proportion of cases is caused by alterations in non-coding regions such as the intronic parts of splice sites. The pathogenicity of these mutations can often not be predicted as easily as alterations leading to classical nonsense or missense situations. Consequently, biochemical workup of the consequences of the sequence changes play an important role in the characterization of a novel mutation. Furthermore, even though chromophobe RCC is most common renal carcinoma entity found in BHD syndrome our case shows that other subtypes are possible and may be associated with a much more aggressive course of disease. Rescuing functional consequences of the mutations involved like altered localization through inhibition of ubiquitination may facilitate future therapeutic strategies to this familial disease.

## Additional files

Additional file 1: Figure S1. Histopathological analysis of the kidney tumor tissue and histopathological work up of the peritoneal metastasis. **a** and **b** Histopathological analysis reveals chromophobe carcinoma in the kidney (a: bar = 1000 μm; b: bar = 50 μm). **c** Beside the nodular growing chromophobe carcinoma (right side) a second tumor component consisting of very small tumor cells can be detected (on the left side of the panel, bar = 1000 μm). **d** and **e** HE staining shows that the peritoneal metastasis consists of the small tumor cell component that was also found in the tumor tissue of the kidney (**d**: bar = 1000 μm; **e**: bar = 50 μm) **f** and **g** Ki-67 staining reveals a very high proliferation index in the small cell component of the peritoneal metastasis (**f**) as well as the small cell component of the kidney tumor (**g**) (**f**: bar = 50 μm, **g**: bar = 100 μm). (PDF 468 kb)

Additional file 2: Figure S2. Exon 10, intron 10 and exon 11 of *FLCN* were cloned into an expression vector. After transfection into mIMCD cells (of mouse origin) RNA and afterwards cDNA was prepared and subjected to sequencing and PCR. As expected in the wildtype (WT) situation, intron 10 (= 565 bp) is spliced out, resulting in a PCR product of 197 bp when using primers that are located in exon10 and exon11. The patient mutation leads to a not functional splice acceptor site in front of exon 11. As a consequence, intron 10 is not spliced out leading to larger PCR product of 762 bp. Human cDNA and human genomic DNA served as controls for the PCR products. Sanger sequencings confirmed the results, electropherograms of the crucial junctions are shown. (PDF 929 kb)

Additional file 3: Figure S3. **a** Western blots were loaded with whole cell lysates of cultured human cells (RPE-1) that had been transfected either with scrambled control siRNA or with two different siRNAs targeting FLCN. Staining with the anti-FLCN antibody shows one specific band at the expected molecular weight the intensity of which is strongly reduced by FLCN knockdown (left panel). Anti-Actin staining of the same membrane was used to control for equal loading (right panel). **b** To check whether the antibody was suitable for IHC as well we stained cell pellets of the established FLCN knockout cell line UOK257 lentivirally transduced to express emGFP (left image) showing little to no signal. UOK257cells that had been lentivirally transduced to express FLCN show a strong signal (magnification 40×). **c** IHC in human tissue shows that FLCN can still be detected in the chromophobe renal cell carcinoma of the BHD patient (middle panel). Normal human kidney tissue from a control (left side) was stained for comparison and shows a stronger signal. A control staining without the FLCN first antibody is shown on the right side of the panel (magnification 40×). (PDF 9710 kb)

## Abbreviations

AAVS1: Adeno-associated virus integration site 1
AMPK: AMP-activated protein kinase
array CGH: Array comparative genomic hybridization
BHD: Birt-Hogg-Dubé
ccRCC: Clear-cell RCC
cDNA: Complementary DNA
CDX2: Caudal type homeobox transcription factor 2
chRCC: Chromophobe RCC
CK7: Cytokeratin 7
CKAE1/3: Pan cytokeratin
CT: Computed tomography
DNA: Deoxyribonucleic acid
FLCN: Folliculin
FNIP: Folliculin-interacting protein
fp: Forward primer
GFP: Green fluorescent protein
HCl: Hydrochloric acid
HEK: Human embryonic kidney cells
HGMD: Human gene mutation database
HIF: Hypoxia-inducible transcription factor
IHC: Immunohistochemistry
IMCD: Inner medullary collecting duct cells
Ki-67: Marker of proliferation Ki-67
LOVD: Leiden open variation database
MLPA: Multiplex ligation-dependent probe amplification
mTOR: Mechanistic target of rapamycin
Mut: Mutant
PAGE: Polyacrylamide gel electrophoresis
PCR: Polymerase chain reaction
PFA: Paraformaldehyde
PVDF: Polyvinylidene fluoride
RCC: Renal cell carcinoma
RNA: Ribonucleic acid
rp: Reverse primer
RPE-1: Retina pigmented epithelium cells
SDS: Sodium dodecyl sulfate
siRNA: Short interfering RNA
TALEN: Transcription activator-like effector nuclease
TGFb: Transforming growth factor beta
TTF-1: Thyroid transcription factor 1
VEGF: Vascular endothelial growth factor
VHL: Von-Hippel-Lindau
WT: Wildtype
