# Supplementary figures and images for: Characterization of a splice-site mutation in the tumor suppressor gene FLCN associated with renal cancer
Source: BMC Med Genet. 2017 May 12;18:53. doi: 10.1186/s12881-017-0416-5 (PMC5429543; doi:10.1186/s12881-017-0416-5)

a

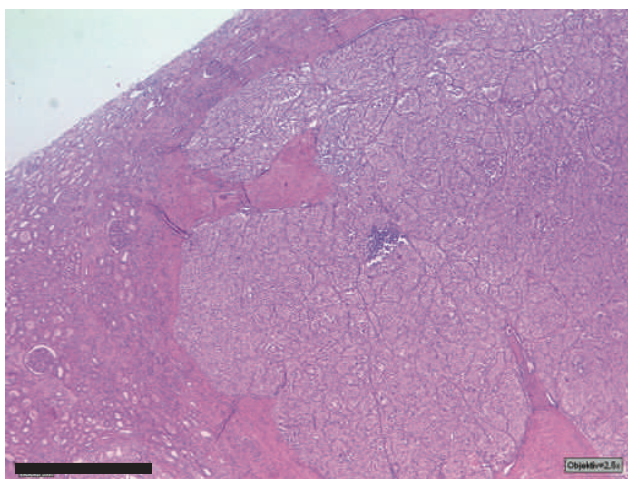

b

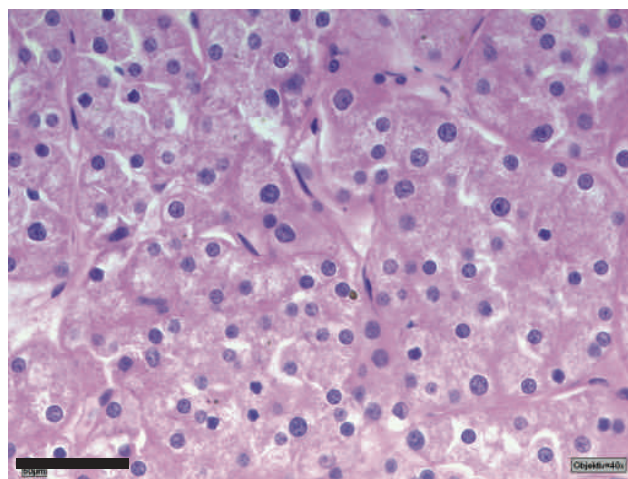

c

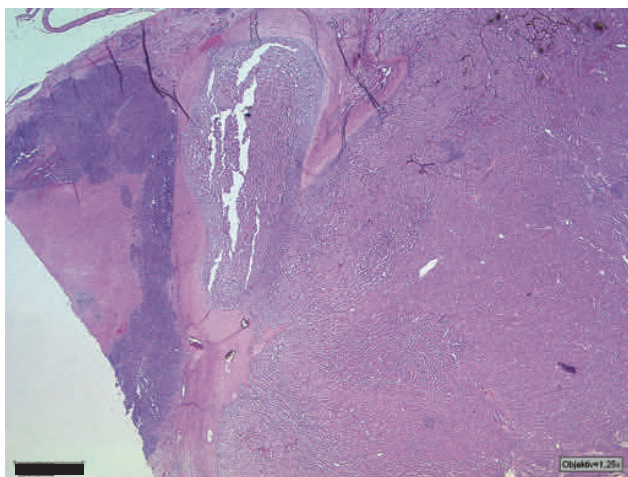

d

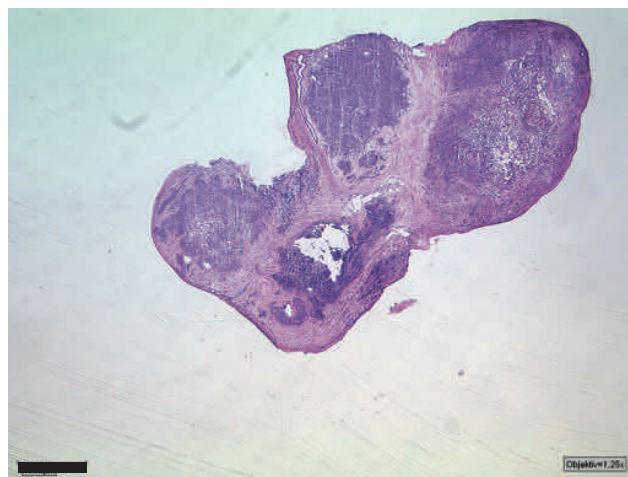

e

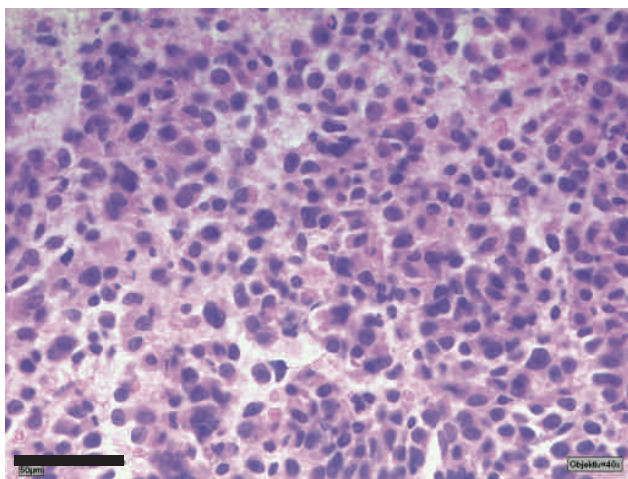

f

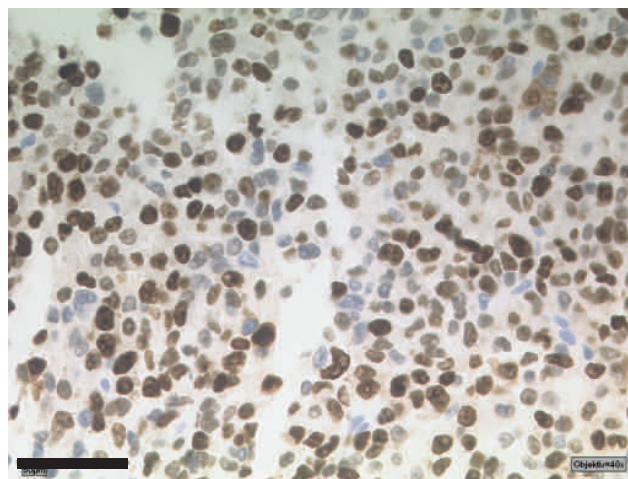

g

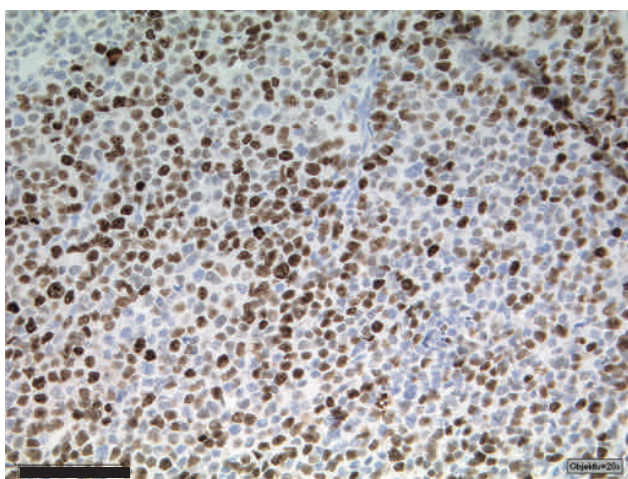

Supplementary Figure 1

Supplement: Supplementary file 1 — Histopathological analysis of the kidney tumor tissue and histopathological work up of the peritoneal metastasis. a and b Histopathological analysis reveals chromophobe carcinoma in the kidney (a: bar = 1000 μm; b: bar = 50 μm). c Beside the nodular growing chromophobe carcinoma (right side) a second tumor component consisting of very small tumor cells can be detected (on the left side of the panel, bar = 1000 μm). d and e HE staining shows that the peritoneal metastasis consists of the small tumor cell component that was also found in the tumor tissue of the kidney (d: bar = 1000 μm; e: bar = 50 μm) f and g Ki-67 staining reveals a very high proliferation index in the small cell component of the peritoneal metastasis (f) as well as the small cell component of the kidney tumor (g) (f: bar = 50 μm, g: bar = 100 μm). (PDF 468 kb) [file 12881_2017_416_MOESM1_ESM.pdf]

minigene-1

WT

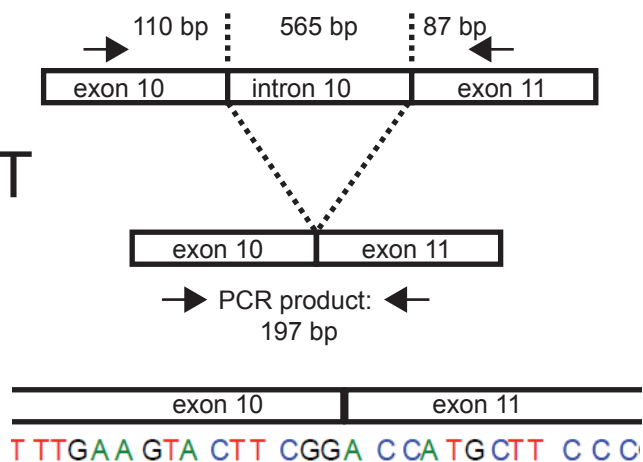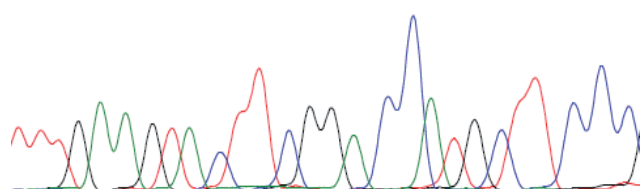

Mut

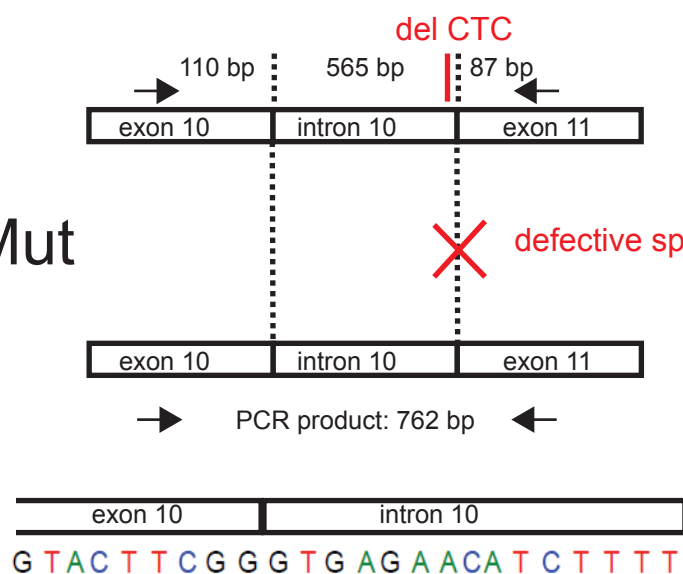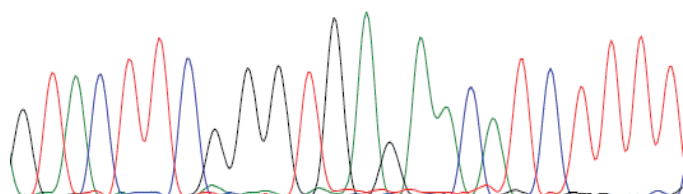

minigene-1 WT  
minigene-1 Mut  
H2O  
human cDNA  
human genomic DNA

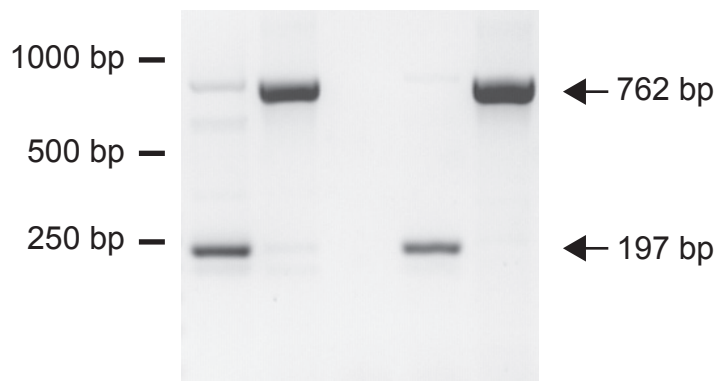

Supplementary Figure 2

Supplement: Supplementary file 2 — Exon 10, intron 10 and exon 11 of FLCN were cloned into an expression vector. After transfection into mIMCD cells (of mouse origin) RNA and afterwards cDNA was prepared and subjected to sequencing and PCR. As expected in the wildtype (WT) situation, intron 10 (= 565 bp) is spliced out, resulting in a PCR product of 197 bp when using primers that are located in exon10 and exon11. The patient mutation leads to a not functional splice acceptor site in front of exon 11. As a consequence, intron 10 is not spliced out leading to larger PCR product of 762 bp. Human cDNA and human genomic DNA served as controls for the PCR products. Sanger sequencings confirmed the results, electropherograms of the crucial junctions are shown. (PDF 929 kb) [file 12881_2017_416_MOESM2_ESM.pdf]
