# Supplementary material for: Characterization of a splice-site mutation in the tumor suppressor gene FLCN associated with renal cancer
Source: BMC Med Genet. 2017 May 12;18:53. doi: 10.1186/s12881-017-0416-5 (PMC5429543; doi:10.1186/s12881-017-0416-5)
Supplement: Supplementary file 3 — a Western blots were loaded with whole cell lysates of cultured human cells (RPE-1) that had been transfected either with scrambled control siRNA or with two different siRNAs targeting FLCN. Staining with the anti-FLCN antibody shows one specific band at the expected molecular weight the intensity of which is strongly reduced by FLCN knockdown (left panel). Anti-Actin staining of the same membrane was used to control for equal loading (right panel). b To check whether the antibody was suitable for IHC as well we stained cell pellets of the established FLCN knockout cell line UOK257 lentivirally transduced to express emGFP (left image) showing little to no signal. UOK257cells that had been lentivirally transduced to express FLCN show a strong signal (magnification 40×). c IHC in human tissue shows that FLCN can still be detected in the chromophobe renal cell carcinoma of the BHD patient (middle panel). Normal human kidney tissue from a control (left side) was stained for comparison and shows a stronger signal. A control staining without the FLCN first antibody is shown on the right side of the panel (magnification 40×). (PDF 9710 kb) [file 12881_2017_416_MOESM3_ESM.pdf]

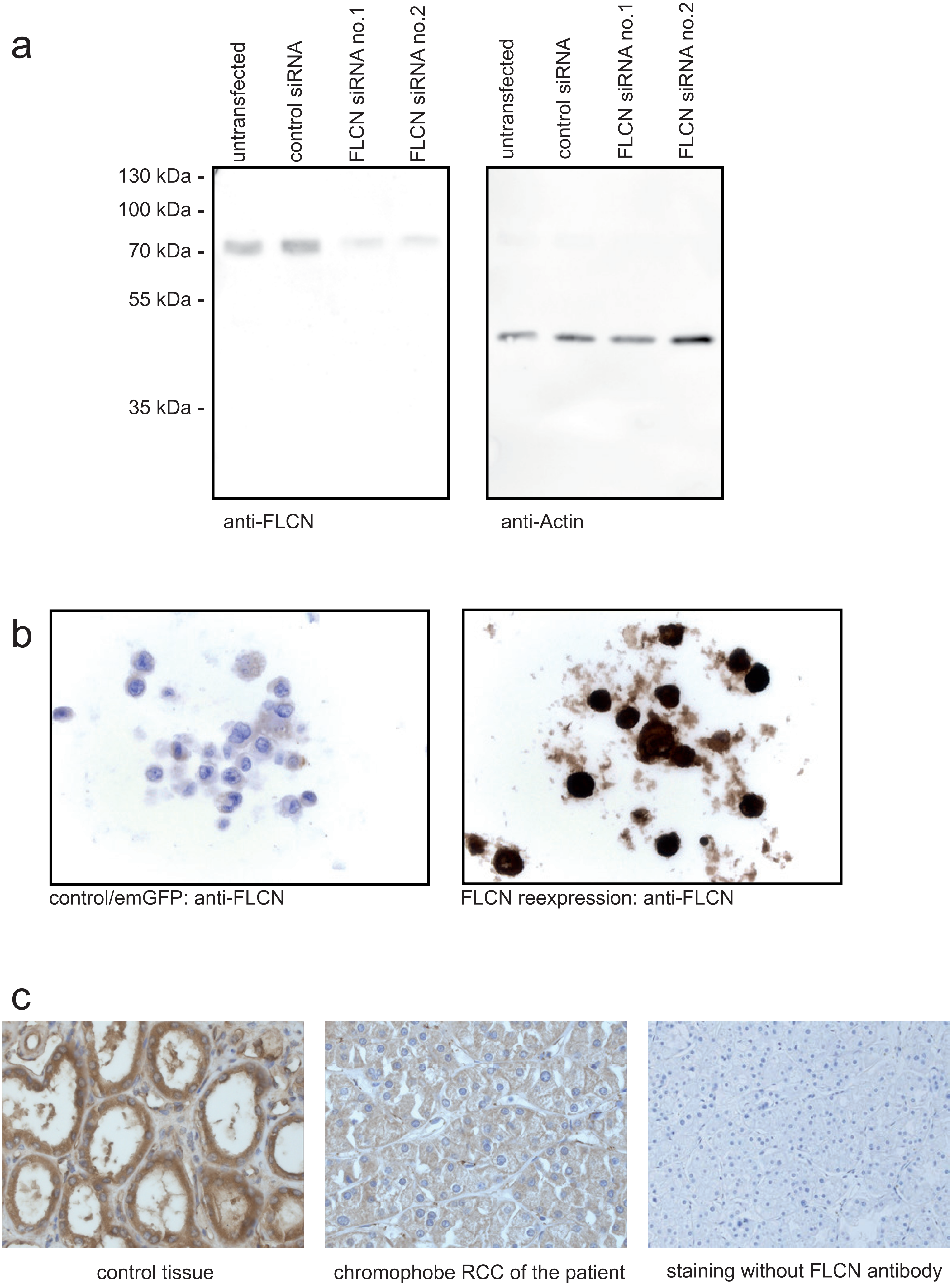

Supplementary Figure 3
